# Dose-related Effects of Calcium to Enhance the Effects of L-tryptophan on Gut Hormones and Energy Intake in Obesity

**DOI:** 10.1210/clinem/dgaf008

**Published:** 2025-01-09

**Authors:** Javad Anjom-Shoae, Penelope C E Fitzgerald, Michael Horowitz, Jens J Holst, Jens F Rehfeld, Simon Veedfald, Christine Feinle-Bisset

**Affiliations:** Adelaide Medical School, University of Adelaide, Adelaide, SA 5005, Australia; Centre of Research Excellence in Translating Nutritional Science to Good Health, University of Adelaide, Adelaide, SA 5005, Australia; Adelaide Medical School, University of Adelaide, Adelaide, SA 5005, Australia; Centre of Research Excellence in Translating Nutritional Science to Good Health, University of Adelaide, Adelaide, SA 5005, Australia; Adelaide Medical School, University of Adelaide, Adelaide, SA 5005, Australia; Centre of Research Excellence in Translating Nutritional Science to Good Health, University of Adelaide, Adelaide, SA 5005, Australia; Endocrine and Metabolic Unit, Royal Adelaide Hospital, Adelaide, SA 5005, Australia; Department of Biomedical Sciences, University of Copenhagen, DK-2200 Copenhagen, Denmark; The Novo Nordisk Foundation Centre for Basic Metabolic Research, University of Copenhagen, DK-2200 Copenhagen, Denmark; Department of Clinical Biochemistry, Rigshospitalet, DK-2100 Copenhagen, Denmark; Department of Biomedical Sciences, University of Copenhagen, DK-2200 Copenhagen, Denmark; Adelaide Medical School, University of Adelaide, Adelaide, SA 5005, Australia; Centre of Research Excellence in Translating Nutritional Science to Good Health, University of Adelaide, Adelaide, SA 5005, Australia

**Keywords:** amino acid, cholecystokinin, food intake, glucagon-like peptide-1, gut functions, peptide tyrosine tyrosine

## Abstract

**Context:**

In males of normal weight, intraduodenal administration of calcium enhances the effects of the amino acid L-tryptophan (Trp) to suppress energy intake, associated with greater stimulation of cholecystokinin (CCK), glucagon-like peptide-1 (GLP-1), and peptide tyrosine-tyrosine (PYY) secretion (key mechanisms underlying the regulation of pyloric motility and gastric emptying) but not gastrin or glucose-dependent insulinotropic polypeptide (GIP).

**Objective:**

Given the implications for the management of obesity, the current study evaluated the effects of calcium, when administered alone and in combination with Trp, on gut hormone secretion, antropyloroduodenal motility, and energy intake in males with obesity.

**Methods:**

Fifteen males with obesity and without type 2 diabetes (mean ± SD; age: 27 ± 8 years; body mass index: 30 ± 2 kg/m^2^; hemoglobin A1c: 5.3 ± 0.2%), received 150-minute intraduodenal infusions of 0, 500, or 1000 mg calcium, each combined with Trp (load: 0.1 kcal/min, known to have submaximal energy-intake suppressant effects) from t = 75-150 minutes, on 3 separate occasions, in a randomized, double-blind, cross-over order. Plasma concentrations of gastrin, CCK, GIP, GLP-1, PYY, and pyloric pressures were measured during the infusions. Immediately postinfusion (t = 150-180 minutes), energy intake at a standardized buffet-style lunch was quantified.

**Results:**

Calcium, in a dose of 1000 mg, stimulated GLP-1, PYY, and pyloric pressures alone (all *P* < .05) and enhanced the effects of Trp to stimulate CCK, GLP-1, and PYY (all *P* < .05), associated with greater suppression of energy intake (*P* = .01). Energy intake (R = −0.64; *P* = .001) was inversely related to the dose of calcium, while plasma concentrations of CCK (R = 0.44; *P* = .05), GLP-1 (R = 0.60; *P* = .01), and PYY (R = 0.83; *P* = .01) were directly related.

**Conclusion:**

Intraduodenal calcium enhances the effect of intraduodenal Trp to stimulate CCK, GLP-1, and PYY and suppress energy intake in males with obesity.

Obesity and its complications, particularly type 2 diabetes, are major global public health challenges, with their prevalence increasing at an alarming rate worldwide (1). This situation reflects, in part, the limitations of existing therapeutic approaches (ie, suboptimal efficacy, high cost, and/or poor long-term tolerability) (2, 3). In considering dietary approaches, the amino acid L-tryptophan (Trp) is of particular interest in appetite regulation, as it is a precursor for the anorexigenic neurotransmitter serotonin (5-hydroxytryptamine) (4-6). Trp is also a potent stimulus of a number of gut hormones and functions that are involved in the regulation of appetite and postprandial blood glucose, including gastric emptying (7). Gut hormones of potential major importance include gastrin, cholecystokinin (CCK), glucose-dependent insulinotropic polypeptide (GIP), glucagon-like peptide-1 (GLP-1), and peptide tyrosine-tyrosine (PYY) (8-10). We have reported that Trp, when administered directly into the stomach (as a bolus dose of 3.3 g) (11) or small intestine (as an infusion in a load of 0.15 kcal/min over 90 minutes) (12), has potent effects to stimulate CCK and GLP-1 secretion in healthy individuals, associated with substantial suppression of energy intake. In these studies, strong inverse relationships between energy intake and circulating concentrations of Trp were evident (11, 12), suggesting that postabsorptive mechanisms may be important.

While not evaluated in humans, there is evidence from preclinical studies that the calcium-sensing receptor (CaSR), where calcium is the major ligand, plays a key role in Trp-induced gut hormone secretion (13-16). We recently reported in males of normal weight that intraduodenal calcium, in a dose of 1000 mg, markedly stimulates GLP-1, PYY, and pyloric pressures and enhances the effects of Trp (in a load of 0.1 kcal/min known to have submaximal effects) to stimulate CCK, GLP-1, and PYY, associated with a substantial reduction in energy intake (17). Calcium unexpectedly reduced the effects of Trp on GIP secretion, which may potentially reflect differences in the mechanisms underlying intestinal nutrient sensing. Plasma concentrations of gastrin were not affected, suggesting minimal, if any, interaction of calcium or Trp with the stomach.

There is a substantial body of evidence, derived from both preclinical and clinical studies, that gastrointestinal (GI) sensitivity to nutrients, particularly lipids, is diminished in obesity, as reflected by attenuated stimulation of appetite-regulatory hormones, including CCK and PYY, and disordered regulation of energy intake (18-21). For example, the effects of intraduodenal oleic acid to stimulate CCK, PYY, and pyloric pressures are diminished in individuals with obesity, compared with those with normal weight, associated with greater energy intake (19). It is, accordingly, important to determine whether the effects of calcium and Trp to stimulate CCK, GLP-1, and PYY secretion and pyloric pressures and suppress energy intake are maintained in individuals with obesity. A positive outcome would have important implications for the development of a novel, nutrient-based approach to management.

The current study, accordingly, aimed to determine the effects of intraduodenal calcium, administered alone and combined with Trp on the stimulation of gut hormone secretion, antropyloroduodenal motility, and energy intake in males with obesity.

## Methods

### Participants

**Figure 1.**
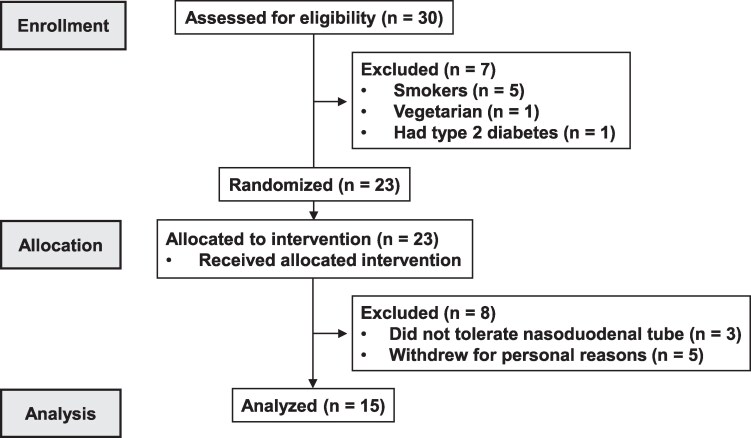
CONSORT flow diagram.

Fifteen adult males with obesity and without type 2 diabetes (mean ± SD; age: 27 ± 8 years; body mass index: 30 ± 2 kg/m^2^; hemoglobin A1c: 5.3 ± 0.2%; n = 11 Caucasians and n = 4 Asians) were included in the study. The participant flow through the study is summarized in Fig. 1. Participants were recruited through flyers placed on the campuses of Adelaide universities, as well as online classified advertisements (University of Adelaide and Gumtree websites). Participants were required to be weight-stable (ie, <5% fluctuation) and to not have adhered to any dietary restriction in the 3 months preceding the study. They were screened and excluded if they had a history of GI disorders or symptoms; had a history of cardiovascular or respiratory diseases or surgery; used supplements or medications known to affect GI functions and/or appetite; consumed protein supplements or >2 standard drinks (20 g) of alcohol >5 days a week; or were lactose intolerant, vegetarian, smokers, or high-performance athletes. The study protocol was approved by the Human Research Ethics Committee of the Central Adelaide Local Health Network and performed in accordance with the Declaration of Helsinki and the National Health and Medical Research Council Statement on Ethical Conduct in Human Research. All participants provided written, informed consent before their inclusion, and after enrollment, each was assigned to a treatment order of balanced randomization that was generated with an online tool (www.randomization.com) by a research officer who was not involved in data analysis. The study was registered as a clinical trial with the Australian and New Zealand Clinical Trials Registry (www.anzctr.org.au; ACTRN12622000739718).

### Study Outline

The study design was identical to our previous study in males of normal weight (17). The study evaluated the effects of 150-minute intraduodenal infusions of 0 mg (Ca-0), 500 mg (Ca-500), or 1000 mg (Ca-1000) calcium, corresponding to calcium alone during infusion period 1 (t = 0-75 minutes), which was then combined, during infusion period 2, with Trp (Ca + Trp) in a load of 0.1 kcal/min, from t = 75-150 minutes (ie, Ca-0 + Trp, Ca-500 + Trp, and Ca-1000 + Trp) on plasma concentrations of gut hormones (ie, gastrin, CCK, GIP, GLP-1, and PYY), antropyloroduodenal motility, appetite perceptions, and ad libitum energy intake. The higher calcium dose was based on the recommended daily intake of calcium (22) and the lower dose to allow the assessment of potential dose-related effects. The load of Trp (0.1 kcal/min) was based on our previous study, in which intraduodenal Trp in a load of 0.15 kcal/min, but not 0.075 kcal/min, suppressed energy intake and stimulated gut hormones in healthy males (12) and thus represented a submaximal load. Intraduodenal infusion was used to allow the standardized administration of these treatments, avoiding the potential influence of interindividual variations in the rate of gastric emptying and allowing small intestinal mucosal receptors and nutrient transporters to be targeted directly without the influence of cephalic stimuli (taste, swallowing, smell).

### Preparation of Study Treatments

On each study day, 2 solutions were prepared: 1 for infusion period 1 (t = 0-75 minutes), consisting of calcium alone, and 1 for infusion period 2 (t = 75-150 minutes), containing calcium and Trp, as described (17). All solutions were isosmotic (300mOsm), prepared on the morning of the study and infused at a rate of 3 mL/min. The solutions had an identical appearance. Both the investigator performing the study and the participants were blinded to the nature of the infusions.

### Study Protocol

Each participant was studied on 3 occasions, separated by 3 to 7 days, in a randomized, double-blind, cross-over order. Participants were instructed to maintain their usual eating habits between study days and refrain from strenuous exercise and alcohol for 24 hours before each study and were provided with a standardized meal (beef lasagne, McCain Food, Wendouree, Victoria, Australia; energy content: 2522 kJ) to be consumed in full with only water between 5.30 Pm and 6 Pm the night prior to each study visit. Participants were asked to abstain from any other food and drinks, except water (which was allowed until 7 Am), until they attended the Clinical Research Facility at 8 Am the next morning. On arrival, a nasoduodenal catheter (outer diameter: 3.5 mm, total length: 100 cm; Dentsleeve International, Mui Scientific, Ontario, Canada) (23) for continuous monitoring of antropyloroduodenal motility was inserted into the stomach through an anesthetized nostril and allowed to pass into the duodenum by peristalsis. The catheter was positioned using the sleeve sensor straddling the pylorus (23). Once the catheter was positioned correctly, an intravenous cannula was placed in a forearm vein for blood sampling. Fasting motility was monitored until the occurrence of phase III activity of the fasting migrating motor complex. During the subsequent period of motor quiescence (phase I), a 15-minute baseline recording was taken (ie, at t = −15 to 0 minutes). At t = −15 and 0 minutes, baseline blood samples were collected and the participant completed a visual analog scale (VAS) questionnaire (24) to assess appetite-related perceptions (fullness and hunger), GI symptoms (nausea and bloating), and drowsiness, and then 1 of the solutions for infusion period 1 (ie, t = 0-75 minutes) commenced, followed by the solution for infusion period 2 (ie, t = 75-150minutes). Antropyloroduodenal pressures were recorded continuously for 150 minutes (t = 0-150 minutes), blood samples for measurements of plasma hormones were collected, and VAS questionnaires were completed, every 15 minutes. At t = 150 minutes, the manometric catheter was removed and participants were presented with a standardized, buffet-style meal (25) and instructed to consume as much food as they wished until they felt comfortably full, for up to 30 minutes (t = 150-180 minutes). At t = 180 minutes, a final blood sample and VAS questionnaire were collected. The intravenous cannula was then removed, and participants could then leave the laboratory.

### Measurements

#### Plasma GI hormone concentrations

Blood samples were collected into ice-chilled tubes containing EDTA. Plasma was obtained by centrifugation at ∼1832 *g* force for 15 minutes at 4 °C within 15 minutes of collection and stored at −80 °C until subsequent analysis.

Plasma concentrations of gastrin (pmol/L) and CCK (pmol/L) were measured by in-house radioimmunoassays (RIAs), using a C-terminal specific antibody (no. 2604, RRID: AB_2893010) for gastrin and an antiserum (no. 92128, RRID: AB_2893008) for CCK, which bind the circulating bioactive forms of gastrin and CCK with equal potency, respectively, without cross-reactivity with each other (26, 27).

Total plasma GIP and GLP-1 concentrations (pmol/L) were analyzed by RIAs using a C-terminal directed antiserum (no. 80867, RRID: AB_2892194) for GIP (28) and an antibody (no. 89390, RRID: AB_2892195) with an absolute requirement for the intact amidated C-terminus of GLP-1, which measures both intact and N-terminally truncated forms of GLP-1 (29).

Plasma PYY concentrations (pmol/L) were measured by an in-house RIA using an antiserum (RRID: AB_2895649) against human PYY (1-36) (Sigma-Aldrich, St. Louis, MO, USA) (30).

The detection limit for all RIAs was below 1 pmol/L, and intra- and interassay coefficients of variation were below 10%.

#### Antropyloroduodenal pressures

Antropyloroduodenal pressures were digitized and recorded using a computer-based system running commercially available software (MMS Database software, version 8.17; Solar GI; Laborie Medical Technologies Corp., Portsmouth, NH, USA), as described (23, 31).

#### Appetite perceptions, GI symptoms, and drowsiness

Appetite perceptions, GI symptoms, and drowsiness were quantified using validated 100-mm VAS questionnaires (24).

#### Energy intake

Energy intake (kJ) was calculated from the amount of foods/liquids (g) consumed at the buffet-style meal, quantified by weighing each item before and after being offered to the participant (17). Energy intake (kJ) was then calculated using commercially available software (Foodworks 8.0, Xyris Software, Highgate Hill, QLD, Australia) (25).

### Data and Statistical Analysis

Based on our previous study of the effects of intraduodenal Trp on plasma CCK (12), we calculated that 14 participants would allow the detection of a difference of 1.0 pmol/L, with an SD of 0.8 pmol/L at α = 0.016 with a power of 80%.

Baseline (0) values for all data were calculated as the mean of values obtained at t = −15 minutes and 0 minutes. Total numbers and mean amplitudes of antral and duodenal pressure waves were used to calculate antral and duodenal motility indices using the following equation (32):


$$\begin{array}{rl} & \mathrm{Motility}\;\mathrm{index}\;(\mathrm{mmHg})\;=\\ & \;\mathrm{natural}\;\mathrm{logarithm}[\mathrm{sum}\;\mathrm{of}\;\mathrm{amplitudes}\;\mathrm{timesnumber}\;\\ & \;of\;pressure\;waves]+1. \end{array}$$

Plasma hormone concentrations, VAS scores, the total number and amplitude of isolated pyloric pressure waves, and motility indices of antral and duodenal waves were analyzed separately for infusion period 1 (ie, t = 0-75 minutes), to quantify the effects of calcium alone, and infusion period 2 (ie, t = 75-150 minutes), to determine whether calcium enhanced the effects of Trp. Data were analyzed using restricted maximum likelihood mixed-effects models with treatment, time, and the treatment × time interaction as fixed effects; baseline values as a fixed covariate; and an unstructured covariance matrix to account for the repeated visits per participant. Energy intake and the amount of food consumed at the buffet meal were also analyzed using restricted maximum likelihood mixed-effects models, with treatment as a fixed effect and an unstructured covariance matrix to account for the repeated visits per participant. Post hoc comparisons, adjusted for multiple comparisons by Bonferroni correction, were performed when significant main effects or interactions were found. Comparisons between postmeal (t = 180 minutes) and premeal (t = 150 minutes) values were performed using a paired *t* test. Relationships between energy intake with plasma hormone concentrations at t = 150 (ie, immediately before the buffet meal), calcium dose, and total number and mean amplitude of isolated pyloric pressure waves, and between plasma hormone concentrations at t = 0-75 minutes and t = 75-150 minutes [each expressed as area under the curve (AUCs) (calculated using the trapezoidal rule), ie, AUC_0-75 minutes_ and AUC_75-150 minutes_] and calcium dose were evaluated using linear within-subject correlations. Statistical analysis was performed using SPSS software (version 28.0; IBM, Chicago, IL, USA). Differences were considered statistically significant at *P* ≤ .05. All data are reported as means with SEMs.

## Results

All participants completed all study days and tolerated the study protocol well.

### Plasma Hormone Concentrations

There were no differences in basal values between study days for plasma gastrin, CCK, GIP, GLP-1, or PYY.

#### Gastrin

**Figure 2.**
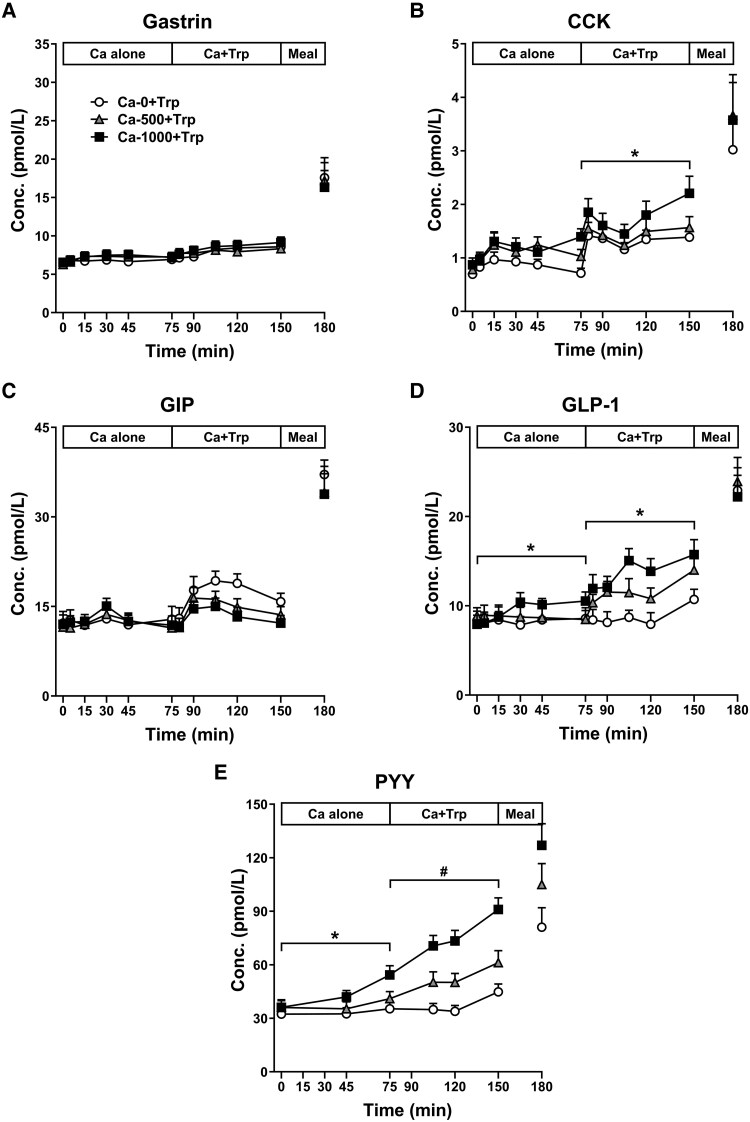
Plasma gastrin (A), CCK (B), GIP (C), GLP-1 (D), and PYY (E) concentrations during 150-minute intraduodenal infusions of 0 mg (Ca-0), 500 mg (Ca-500), or 1000 mg (Ca-1000) calcium, corresponding to calcium alone (Ca alone) during infusion period 1 (t = 0-75 minutes), which was then combined, during infusion period 2, with Trp (Ca + Trp), in a load of 0.1 kcal/min, from t = 75-150 minutes (ie, Ca-0 + Trp, Ca-500 + Trp, and Ca-1000 + Trp) and after a buffet-style meal at t = 150-180 minutes. Data are expressed as mean ± SEM; n = 15. Data were analyzed using restricted maximum likelihood mixed-effects models, with treatment, time, and the treatment × time interaction as fixed effects; baseline values as a fixed covariate; and an unstructured covariance matrix to account for the repeated visits per participant. *Treatment effect, *P* < .05; #Treatment × time interaction, *P* < .05. Abbreviations: Ca, calcium; CCK, cholecystokinin; Conc, concentration; GIP, glucose-dependent insulinotropic polypeptide; GLP-1, glucagon-like peptide-1; PYY, peptide tyrosine-tyrosine; Trp, L-tryptophan.

There was no effect of treatment for either calcium alone or calcium + Trp (Fig. 2A). Plasma gastrin increased after the meal on each day and was higher at t = 180 minutes, compared with t = 150 minutes (all *P* < .01), with no differences between days.

#### CCK

There was no effect of treatment for calcium alone (Fig. 2B). However, there was an effect of treatment for calcium + Trp (*P* = .04). Ca-1000 + Trp increased plasma CCK, compared with Ca-0 + Trp (*P* = .04), while Ca-500 + Trp had no effect (Fig. 2B). Plasma CCK increased after the meal on each day and was higher at t = 180 minutes, compared with t = 150 minutes (all *P* < .05), with no differences between treatments.

#### GIP

There was no effect of treatment for calcium alone (Fig. 2C) and a trend for an effect of treatment for calcium + Trp (*P* = .09). Plasma GIP levels tended to be lower in response to both Ca + Trp-500 and Ca + Trp-1000, compared with Ca-0 + Trp (Fig. 2C). Plasma GIP increased after the meal on each day and was higher at t = 180 minutes, compared with t = 150 minutes (all *P* < .05), with no differences between treatments.

#### GLP-1

There was a treatment effect for calcium alone (*P* = .02) (Fig. 2D). Ca-1000 increased plasma GLP-1, compared with both Ca-500 (*P* = .03) and Ca-0 (*P* = .03), while Ca-500 had no effect. There was also a treatment effect for calcium + Trp (*P* = .001) (Fig. 2D). Both Ca-1000 + Trp (*P* = .001) and Ca-500 + Trp (*P* = .03) increased plasma GLP-1, compared with Ca-0 + Trp. Plasma GLP-1 increased after the meal on each day and was higher at t = 180 minutes, compared with t = 150 minutes (all *P* < .05), with no differences between treatments.

#### PYY

There was a treatment effect for calcium alone (*P* = .001) (Fig. 2E). Ca-1000 increased plasma PYY, compared with both Ca-500 (*P* = .002) and Ca-0 (*P* = .002), while Ca-500 had no effect. There was a treatment × time interaction for calcium + Trp (*P* = .002) (Fig. 2E). Ca-500 + Trp increased plasma PYY at t = 105 and 120 minutes, compared with Ca-0 + Trp (all *P* < .05). Ca-1000 + Trp increased plasma PYY between t = 75-150 minutes, compared with both Ca-500 + Trp and Ca-0 + Trp (all *P* < .05). Plasma PYY increased after the meal on each day and was higher at t = 180 minutes, compared with t = 150 minutes (all *P* < .05), with no differences between treatments.

### Antropyloroduodenal Pressures

Basal values for antral, pyloric, and duodenal pressures did not differ between study days (Supplemental Table 1) (33).

#### Antral pressures

There was no effect of treatment in response to either calcium alone or Ca + Trp (Supplemental Table 1) (33).

#### Isolated pyloric pressure waves

**Figure 3.**
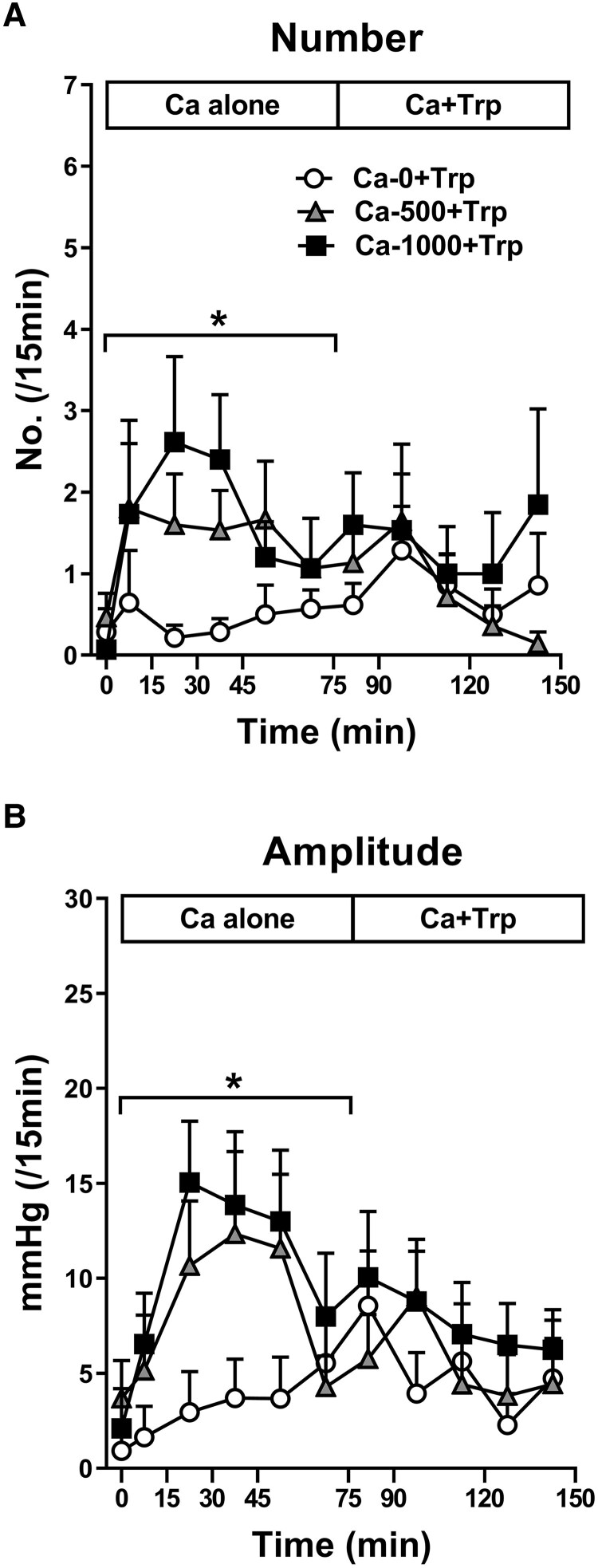
Number (A) and amplitude (B) of isolated pyloric pressure waves during 150-minute intraduodenal infusions of 0 mg (Ca-0), 500 mg (Ca-500), or 1000 mg (Ca-1000) calcium, corresponding to calcium alone (Ca alone) during infusion period 1 (t = 0-75 minutes), which was then combined, during infusion period 2, with Trp (Ca + Trp), in a load of 0.1 kcal/min, from t = 75-150 minutes (ie, Ca-0 + Trp, Ca-500 + Trp, and Ca-1000 + Trp). Data are expressed as mean ± SEM; n = 15. Data were analyzed using restricted maximum likelihood mixed-effects models, with treatment, time, and the treatment × time interaction as fixed effects; baseline values as a fixed covariate; and an unstructured covariance matrix to account for the repeated visits per participant. *Treatment effect, *P* < .05. Abbreviations: Ca, calcium; Trp, L-tryptophan.

There were effects of treatment on both the number (*P* = .01) and amplitude (*P* = .03) of isolated pyloric pressure waves for calcium alone (Fig. 3). Ca-1000 increased, and there was a trend for Ca-500 to increase both the number (*P* = .04, *P* = .08, respectively) and the amplitude (*P* = .02, *P* = .08, respectively) of isolated pyloric pressure waves, compared with Ca-0. There were no further effects of treatment for calcium + Trp (Fig. 3).

#### Duodenal pressures

There was an effect of treatment for calcium alone on duodenal pressures (*P* = .01) (Supplemental Table 1) (33). Ca-1000 reduced the motility index of duodenal pressure waves, compared with Ca-0 (*P* < .05), while Ca-500 had no effect. There was also an effect of treatment for calcium + Trp (*P* = .02) (Supplemental Table 1) (33). Ca-1000 + Trp reduced the motility index of duodenal pressure waves, compared with Ca-0 + Trp (*P* < .05), while Ca-500 + Trp had no effect.

### Appetite Perceptions, GI Symptoms and Drowsiness

There were no differences in baseline scores of appetite perceptions (hunger and fullness), GI symptoms (nausea, bloating), or drowsiness between study days or any effects of treatment during the infusions (Supplemental Fig. 1) (33).

### Energy Intake

**Figure 4.**
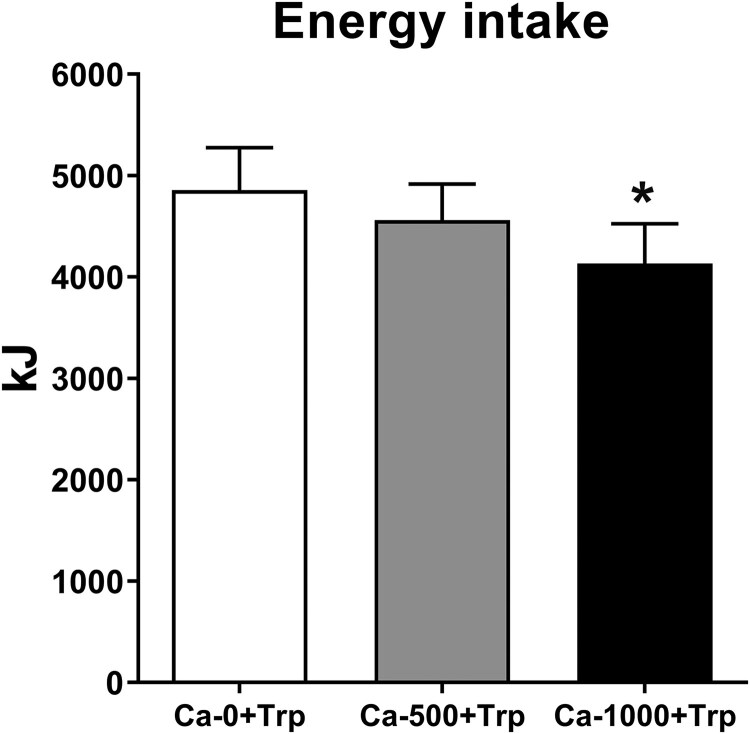
Energy intake at the buffet-style meal following 150-minute intraduodenal infusions of 0 mg (Ca-0), 500 mg (Ca-500), or 1000 mg (Ca-1000) calcium, corresponding to calcium alone (Ca alone) during infusion period 1 (t = 0-75 minutes), which was then combined, during infusion period 2, with Trp (Ca + Trp), in a load of 0.1 kcal/min, from t = 75-150 minutes (ie, Ca-0 + Trp, Ca-500 + Trp, and Ca-1000 + Trp). Data are expressed as mean ± SEM; n = 15. Data were analyzed using restricted maximum likelihood mixed-effects models, with treatment as a fixed effect and an unstructured covariance matrix to account for the repeated visits per participant and adjustment for multiple comparisons made using Bonferroni correction. * *P* < .05 vs control. Abbreviations: Ca, calcium; kJ, kilojoules; Trp, L-tryptophan.

There were effects of treatment on energy intake (*P* = .01) (Fig. 4) and the amount consumed (*P* = .005) at the buffet meal. Ca-1000 + Trp decreased energy intake by 724 ± 176 kJ (∼15%) (*P* = .01), and the amount consumed by 307 ± 62 g, compared with Ca-0 + Trp (*P* = .01), while Ca-500 + Trp had no effect.

### Relationships of Energy Intake With Plasma Hormone Concentrations, Calcium Dose, and Antropyloroduodenal Pressures

Energy intake at the buffet meal correlated inversely with the dose of calcium (R = −0.64; *P* = .001), plasma CCK (R = −0.42; *P* = .05), and PYY (R = −0.47; *P* = .001) concentrations immediately before the meal (ie, at t = 150 minutes) (Supplemental Fig. 2) (33). Plasma concentrations of GLP-1 (R = 0.53; *P* = .02) and PYY (R = 0.57; *P* = .01) from t = 0-75 minutes and of CCK (R = 0.44; *P* = .05), GLP-1 (R = 0.60; *P* = .01), and PYY (R = 0.83; *P* = .01) from t = 75-150 minutes (Supplemental Fig. 3) (33) were related directly to the dose of calcium. There were no other significant relationships between energy intake, other hormones, pyloric pressures, and calcium dose.

## Discussion

Our study demonstrates dose-related effects of intraduodenal calcium to (1) stimulate plasma GLP-1 and PYY, as well as pyloric pressures, and (2) enhance the effects of Trp to stimulate CCK, PYY, and GLP-1, associated with greater suppression of energy intake, in males with obesity. These observations, accordingly, extend the findings of our previous study in individuals of normal weight to those with obesity and encourage therapeutic development of this approach for the management of obesity.

The current study was stimulated by our recent observations in healthy individuals of normal weight that the addition of calcium, in doses of 500 and 1000 mg intraduodenally, increased the Trp-induced suppression of energy intake by 615 kJ and 778 kJ, respectively (17). The rationale for the combination of Trp with calcium was underpinned by preclinical evidence that the CaSR, which has a well-established role in sensing luminal calcium and calcium homeostasis (34), also mediates Trp-induced gut hormone secretion (13-16), central to the suppression of energy intake. We have now demonstrated that these effects of calcium, in identical doses, are evident in males with obesity; that is, both 500- and 1000-mg doses of calcium enhanced the suppression of energy intake by Trp by 293 kJ and 724 kJ, respectively. That the effect of the lower dose was not statistically significant may potentially be attributable to the lack of adjustment of the calcium dose for the body weight of the participants. Nevertheless, these findings support the potential use of calcium, in combination with Trp, to optimize the effects of current dietary approaches to weight loss; moreover, it may have a role in reducing the potential for weight regain following pharmacologically or surgically induced weight loss. Studies are now indicated to determine whether these effects are sustained in the longer term and the feasibility of delivering these nutrients through practical dietary approaches, such as calcium- and Trp-enriched milk-based drinks or encapsulated supplements, to potentially facilitate their future integration into weight-loss strategies.

Consistent with our observations in individuals of normal weight (17), calcium enhanced the effects of Trp to stimulate plasma CCK, GLP-1, and PYY, in a dose-dependent manner, in males with obesity. The concept that the effects of calcium to enhance energy intake suppression by Trp may be attributable to the stimulation of gut hormones is supported by the observation that energy intake was inversely related to plasma concentrations of CCK and PYY before the meal. Interestingly, plasma hormone concentrations immediately after the meal did not differ between treatment days, despite a substantially reduced intake on the Ca-1000 + Trp day. This suggests that in addition to the dietary components of the buffet meal, the prior study treatment contributed to the release of hormones, to enhance the appetite-suppressant effect. Thus, integration (probably at a central level) of the inputs from hormone release in response to the nutrient infusion prior to the meal, hormone release during the meal, as well as other factors, such as pyloric stimulation, is likely to determine the magnitude of energy-intake suppression at the meal.

In relation to the important role of GI hormones in the effects observed in our study, it is important to note that while the observed elevations in plasma hormone concentrations in response to luminal nutrients are likely much lower than those induced by pharmacological agents (eg, GLP-1 receptor agonists), the nutrient-induced concentrations of GI hormones at the site of their release, where they interact locally with receptors on vagal afferents or enteric cells, are likely to be substantially higher (35). This represents a fundamental distinction between the mechanisms underlying the action of nutrient-induced GI hormone release and hormone agonists, with the latter increasing plasma but not local concentrations substantially (an effect that is likely also responsible for the frequent adverse effects, such as nausea) (36). Thus, the observed effects of calcium and Trp on energy intake suppression are likely to reflect local actions of GI hormones in the vicinity of their release and also at more distal sites, brought about by circulating hormone concentrations.

Our study was not designed to determine the luminal mechanisms underlying the effects on GI hormones, but the observed patterns of secretion of these hormones (ie, a prompt increase in plasma CCK but gradual rises in GLP-1 and PYY) may well reflect the luminal regional distribution of proximally located I-cells and more distally located L-cells, which both express the CaSR on their membranes (37). Accordingly, mechanisms triggered by direct interactions of nutrients with intestinal chemoreceptors, including the CaSR, may mediate the observed effects. This said, while our findings align with preclinical studies demonstrating that the interaction of calcium and Trp increases the stimulation of gut hormones via the CaSR, we did not employ a CaSR antagonist in our study and, accordingly, cannot conclusively attribute the observed effects solely to this receptor or exclude the involvement of other receptors.

Consistent with our findings in individuals of normal weight (17), GIP secretion, in contrast to other hormones, tended to be suppressed in response to calcium and Trp. There is no other information about the effects of Trp on GIP secretion, but in most studies that investigated the effects of calcium on GIP, no effects were evident (38-40). Our findings of modest suppression of GIP secretion may suggest that mechanisms underlying gut hormone release differ between K-cells and other enteroendocrine cells. In support of this concept, previous studies have reported that various luminal stimuli (eg, fructose or hypertonic solutions) stimulated GLP-1 but not GIP (41, 42).

That there were no effects on plasma gastrin either after calcium alone or calcium and Trp is consistent with our previous observations (17) but appears to be in contrast to several studies that reported stimulation of gastrin by calcium (43, 44) or Trp (45, 46). However, these studies employed either an oral or intragastric intervention, and gastrin is primarily secreted by gastric G-cells, which also express the CaSR (47).

That calcium, when administered alone, stimulated PYY and GLP-1 but not CCK secretion is consistent with our previous study (17). There is also some, limited clinical evidence that acute and chronic dietary supplementation of calcium (∼1000 mg/day) increases GLP-1 and PYY, while effects on CCK have not been assessed (38, 48, 49). However, in these studies, calcium was administered as a component of a meal or drink, compromising the assessment of the effect of calcium per se.

Pyloric pressures are known to be independent determinants of the reduction in energy intake induced by the direct administration of nutrients into the small intestine (7). In line with our study of normal-weight participants (17), increased stimulation of both the frequency and amplitude of pyloric pressures was evident in response to calcium alone, which may reflect the direct effects of calcium on the pyloric smooth muscle (50) and/or be mediated by the observed stimulation of GLP-1 and PYY secretion (51). However, no effects were evident in response to calcium and Trp, which may be attributable to the inherently nonsustained nature of pyloric pressure responses (12). Nonetheless, the observed stimulation of pyloric pressures by calcium alone may contribute to energy-intake suppression. The stimulation of pyloric contractions activates mechanoreceptors on vagal afferents, which transmit the signal to the brain where it is integrated with other signals (eg, hormones), and together they contribute to the termination of a meal. The stimulation of pyloric pressures is also a key mechanism underlying the slowing of gastric meal emptying, which increases gastric distension and, thereby, fullness (52).

Limitations of our study should be noted. We did not measure plasma Trp levels, which appear to contribute to energy-intake suppression by Trp (11, 12), although, in our previous study, calcium did not affect circulating Trp concentrations. Because our study was deliberately designed to evaluate the effects of calcium alone and then combined with Trp, we did not assess the effects of calcium per se on energy intake or determine whether the observed effects on GI hormones reflected the interaction of calcium with Trp and/or the action of calcium per se. To limit the burden on participants, we did not include a control condition but recognize that this may have provided insights as to whether, and/or to what extent, Trp alone may have suppressed energy intake. It should also be appreciated that sex-specific changes in Trp pathways to regulate appetite and energy intake have been reported (53)—we only included males as they have been shown to be more sensitive to dietary manipulation (18) and to avoid potential confounding effects of the menstrual cycle on gut hormone secretion or appetite (54).

We conclude that, in males with obesity, intraduodenal calcium, when administered alone, stimulates GLP-1 and PYY secretion and pyloric pressures and enhances the effects of Trp to stimulate CCK, GLP-1, and PYY secretion, associated with greater suppression of energy intake. These observations, accordingly, indicate that calcium has the capacity to modulate GI hormonal and motor functions, integral to the regulation of energy intake. Studies are now indicated to determine whether these effects are retained in the longer term, with the potential of novel nutrient-based approaches for the management of obesity.

## Data Availability

The datasets of the current study are not publicly available but are available from the corresponding author upon reasonable request. **Clinical Trail Registration:** The study was registered as a clinical trial with the Australian and New Zealand Clinical Trials Registry (www.anzctr.org.au; ACTRN12622000739718).

## Abbreviations

AUC: area under the curve
CaSR: calcium-sensing receptor
CCK: cholecystokinin
GI: gastrointestinal
GIP: glucose-dependent insulinotropic polypeptide
GLP-1: glucagon-like peptide-1
PYY: peptide tyrosine-tyrosine
RIA: radioimmunoassay
Trp: L-tryptophan
VAS: visual analog scale
